# The Anatomy, Physiology, Pathology, and Treatment of Cancer

**Published:** 1843-11-11

**Authors:** 


*The Anatomy, Physiology, Pathology, and Treatment of Cancer.* By WALTER HAYLE WASLHE, M.D. Professor of Pathological Anatomy in University College, London, etc. etc. *With Additions.* By J. MASON WARREN, M. D., etc. Boston: William D. Ticknor & Company. 1843. 12mo. pp. 351.

THE medical profession of this country is under great obligations to Dr. WARREN for the present republication, which is an admirable and complete monograph on a most important and interesting subjeet. The work is a republication of the article 'Cancer,' by Dr. WALSHE, which appeared in the Cyclopædia of Practical Surgery, about three years since. It is a masterly critical and analytical exposition of our knowledge of this formidable disease. Our limited space will enable us to convey but a very imperfect notion of its value and completeness.

According to Dr. WALSHE, cancer, or carcinoma, is the Genus of the Order Tissues, of the Class of Heterologous Formations, and belonging to the Family of Adventitious Formations, of which there are *three Species*, agreeing anatomically, physiologically, chemically, and pathologically. These three species are encephaloid, schirrus and colloid, and their characteristics are thus exhibited tabularly, by Dr. Walshe:—

| *Encephaloid.* | *Scirrhus.* | *Colloid.* |
|---|---|---|
| Resembles lobulated cerebral matter. | Resembles rind of bacon traversed by cellulo-fibrous septa. | Has the appearance of partieles of jelly inlaid in a regular alveolar bed. |
| Is commonly opaque from its earliest formation. | Has a semi-transparent glossiness. | The contained matter is strikingly transparent. |
| Is of a dead white colour. | Has a clear whitish or bluish yellow tint. | Greenish yellow is its predominant hue. |
| Contains a multitude of minute vessels. | Is comparatively ill supplied with vessels. | (Its vessels have not been sufficiently examined as yet.) |
| Is less hard and dense than scirrhus. | Is exceedingly firm and dense. | The jelley-like *matter* is exceedingly soft; a colloid *mass* is, however, firm and resisting. |
| Is frequently found in the veins issuing from the diseased mass. | Has not been distinctly detected in this situation. | The pultaceous variety has been detected in the veins. |
| The predominant microscopical elements are globular, not always distinctly cellular, and caudate corpuscula. | The main microscopical constituents are juxtaposed nuclear cells; caudate corpuscula do not exist in it. | Is composed of cells in a state of *emboîtement.* |
| Occasionally attains an enormous bulk. | Rarely acquires larger dimensions than an orange. | Observes a mean in this respect. |
| Has been observed in almost every tissue of the body. | Its seat, as ascertained by observation, is somewhat more limited. | Has so far been seen in a limited number cf parts only. |
| Very commonly coexists in several parts or organs of the same subject. | Is not unusually solitory. | Has rarely been met with in more than one organ. |
| Is remarkable for its occasional vast rapidity of growth. | Ordinarily grows slowly. | Grows with a medium degree of rapidity. |
| Is frequently the seat of interstitial hemorrhage and deposition of black or bistre-coloured matter. | Is comparatively rarely the seat of these changes. | |
| When softened into a pulp appears as a dead white or pink opaque matter of creamy consistence. | Resembles, when softened, a yellowish brown semi-transparent gelatinous matter. | Undergoes no visible change of the kind. |
| Subcutaneous tumours are slow to contract adhesion with the skin. | Scirrhus thus situated usually becomes adherent. | |
| Ulcerated encephaloid is frequently the seat of hemorrhage, followed by rapid fungous development. | Scirrhous ulcers much less frequently give rise to hemorrhage, and fungous growths (provided they retain the scirrhous character) are now more slowly and less abundantly developed. | |
| The progress of the disease after ulceration is commonly very rapid. | There is not such a remarkable change in the rate of progress of the disease atter ulceration has set in. | |
| Is the most common form under which secondary cancer exhibits itself. | | |
| Is the species of cancer most frequently observed in young subjects. | Is much less common before puberty. | Has so far been observed in adults only. |

Dr. WALSHE first considers the anatomical, chemical, and microscopical characters, successively, of encephaloid, schirrus, and colloid. The origin, growth, and decay of cancer are most admirably investigated under the head of physiology. The pathology of cancer includes an inquiry into the greater liability of certain organs to cancerous development, etiology, and the duration, frequency, symptoms, diagnosis, and prognosis of cancer. This section is illustrated with numerous tables, and is most valuable. Dr. W. shows the inaccuracy of the commonly received opinion, that the tendency to cancer reaches its maximum between the thirty-fifth and fiftieth years. The mortality, on the contrary, goes on increasing with each succeeding decade, until the eightieth year. The chapter on Therapeia, or treatment, is searching and admirable in spirit and execution.

From one view of Dr. WALSHE's we are forced to dissent. He appears to consider a cancerous tumor, even in its earliest stages, as local evidence of the general vitiation of the system. This point is so intimately linked with the great question of treatment, that any idea so formidably erroneous as that of our author's must not pass unnoticed. Carcinoma cannot, in our opinion, be, in any manner, ranked among the true blood diseases. Observation disproves this assertion. It is, in its origin, the result of an aberration in local nutrition, and is subsequently disseminated by the blood throughout the whole economy. In the commencement the blood is not tainted, and it only subsequently becomes the vehicle for the diffusion of the morbific matter, and in that manner is implicated.

The second part treats of Cancer of Particular Parts, and it is here that we are indebted to Dr. Warren for his additions. In the original essay, the author did not entirely complete his subject, "in order that he might not interfere with what would appear in another part of the work, hereafter to be published under other heads, to which he refers his readers." The American editor has included the omissions, and has added the surgical operations required, thus rendering the work more acceptable to the practical surgeon. This has been done in entire keeping with the remainder of the volume. Dr. W. has added an appendix, which consists of an analysis of M. Tanchou's Statistics on Cancer, published in the 17th No. of the Examiner.

